# G_i_-Coupled GPCR Signaling Controls the Formation and Organization of Human Pluripotent Colonies

**DOI:** 10.1371/journal.pone.0007780

**Published:** 2009-11-10

**Authors:** Kenta Nakamura, Nathan Salomonis, Kiichiro Tomoda, Shinya Yamanaka, Bruce R. Conklin

**Affiliations:** 1 Gladstone Institute of Cardiovascular Disease, San Francisco, California, United States of America; 2 Department of Medicine, University of California San Francisco, San Francisco, California, United States of America; 3 Center for iPS Cell Research and Application, Kyoto University, Kyoto, Japan; 4 Department of Cellular and Molecular Pharmacology, University of California San Francisco, San Francisco, California, United States of America; KU Leuven, Belgium

## Abstract

**Background:**

Reprogramming adult human somatic cells to create human induced pluripotent stem (hiPS) cell colonies involves a dramatic morphological and organizational transition. These colonies are morphologically indistinguishable from those of pluripotent human embryonic stem (hES) cells. G protein-coupled receptors (GPCRs) are required in diverse developmental processes, but their role in pluripotent colony morphology and organization is unknown. We tested the hypothesis that G_i_-coupled GPCR signaling contributes to the characteristic morphology and organization of human pluripotent colonies.

**Methodology/Principal Findings:**

Specific and irreversible inhibition of G_i_-coupled GPCR signaling by pertussis toxin markedly altered pluripotent colony morphology. Wild-type hES and hiPS cells formed monolayer colonies, but colonies treated with pertussis toxin retracted inward, adopting a dense, multi-layered conformation. The treated colonies were unable to reform after a scratch wound insult, whereas control colonies healed completely within 48 h. In contrast, activation of an alternative GPCR pathway, G_s_-coupled signaling, with cholera toxin did not affect colony morphology or the healing response. Pertussis toxin did not alter the proliferation, apoptosis or pluripotency of pluripotent stem cells.

**Conclusions/Significance:**

Experiments with pertussis toxin suggest that G_i_ signaling plays a critical role in the morphology and organization of pluripotent colonies. These results may be explained by a G_i_-mediated density-sensing mechanism that propels the cells radially outward. GPCRs are a promising target for modulating the formation and organization of hiPS and hES cell colonies and may be important for understanding somatic cell reprogramming and for engineering pluripotent stem cells for therapeutic applications.

## Introduction

**Figure 1:**
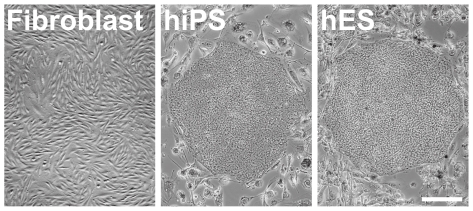
Morphological change during human somatic cell reprogramming. Human dermal fibroblasts undergo a dramatic morphological change during reprogramming and appear to be identical to a colony of human embryonic stem cells. Scale bar, 200 µm.

The ability to reprogram somatic cells back to a pluripotent state has revolutionized our understanding of developmental biology [1]. The creation of patient-specific pluripotent stem cells was a seminal advance toward the use of stem cells for cell-based therapies and regenerative medicine [2]. During reprogramming, adult human somatic cells undergo a remarkable transition from dispersed unicellular fibroblasts to well defined multicellular colonies of human induced pluripotent stem (hiPS) cells that are morphologically indistinguishable from human embryonic stem (hES) cells [2]–[4] (Figure 1). Both hiPS cells and hES cells self-renew indefinitely as highly-organized pluripotent colonies that resemble the inner cell mass from which hES cells are primarily derived [5], [6]. Interestingly, human pluripotent colonies form a flat uniform monolayer, while mouse pluripotent colonies form thicker, multilayered colonies [7]. Since pluripotent colony morphology correlates closely with the maintenance of pluripotency, the mechanisms by which these colonies form and organize may be important for controlling somatic cell reprogramming. Understanding these mechanisms may also allow better control of hiPS cell growth and differentiation *ex vivo* for therapeutic applications.

The molecular mechanisms of human pluripotent colony formation and organization are poorly understood. G protein-coupled receptors (GPCRs) are attractive candidates as modulators of early developmental processes because they are the largest class of cell surface receptors, are the targets of small molecule drugs, and mediate a wide variety of biological processes [8]. Two of the major G-protein families, G_s_ and G_i_, signal through the second messenger cyclic AMP. In particular, G_i_ signaling has emerged as a conserved developmental regulator of cellular morphology, polarity, and migration [9]–[11], including directed chemotaxis of mouse ES cells [12]. The Agtrl1b-Apelin [13], [14], CXCR4-SDF-1 [15], [16] and SPC/S1P-EDG [17], [18] pathways signal using G_i_ and are key mediators of cellular chemotaxis and migration in progenitor and somatic cells. In the self-organizing amoeba *Dictyostehm discoidezlm*, GCPR signaling regulates the formation and organization of multi-cellular colonies by mediating cell-density signaling [19], [20]. In adipocytes, G_i_-coupled signaling regulates cell-density dependent proliferation [21], [22]. The role of G_i_-coupled signaling in pluripotent stem cells is largely unknown, but it has been implicated in the maintenance of pluripotency [23] and directed differentiation [24] of hES cells.

The high number and redundancy among the 370–400 nonordarant GPCRs and G-protein family members poses a challenge for modulating GPCR signaling pathways experimentally [25]. To study the role of GPCR signaling in pluripotent colony morphology and organization, we used bacterial toxins derived from *Bordetella pertussis* and *Vibrio cholerae*, which are global and irreversible modulators of specific G-protein signaling families. Pertussis toxin irreversibly uncouples all commonly expressed members of the G_αi_ gene family (G_αi1_, G_αi2_, G_αi3_, and G_αo_) by ADP-ribosylation at a site that blocks interaction with GPCRs, thereby blocking downstream G_i_-coupled GPCR signaling [26]–[28]. Cholera toxin catalyzes ADP-ribosylation of G_αs_ at a location that constitutively activates G_αs_ and mimics G_s_-coupled GPCR signaling [20], [21].

We hypothesize that hES cell and hiPS cell colonies form and maintain characteristic pluripotent morphology and organization through G_i_-coupled GPCR signaling. To test this hypothesis, we treated pluripotent colonies with pertussis toxin or cholera toxin to irreversibly and specifically modulate G_i_ or G_s_ signaling, respectively, and assessed colony morphology and formation by time-lapse and confocal microscopy.

## Results

### Inhibition of G_i_ Signaling by Pertussis Toxin Prevents Outgrowth of Pluripotent Stem Cell Colonies

**Figure 2:**
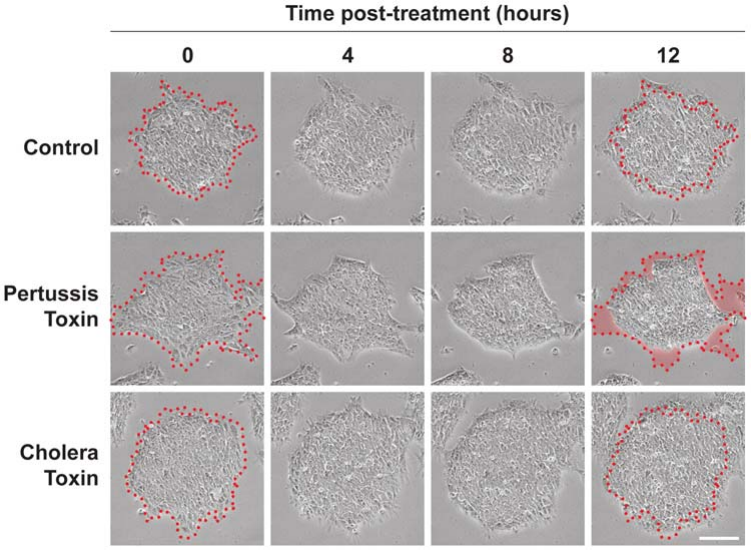
Pertussis toxin induces morphological change in human embryonic stem (hES) cells and human induced pluripotent stem (hiPS) cells. hES cell colony cultured on Matrigel under feeder-free conditions contracts inward, loses pseudopodia-like projections and adopts a dense, aggregated morphology within 12 h after treatment with pertussis toxin. These alterations were not seen in control colonies or colonies treated with cholera toxin treated. Red outline delineates the border of the pluripotent colony at the start of toxin treatment, time 0. The area of colony retraction after 12 h of pertussus toxin treatment is highlighted in red. Scale bar, 200 µm.

To investigate the role of GPCR signaling in pluripotent colony morphology, we first determined the effect of blocking G_i_ signaling in colonies of hES cell or hiPS cells with pertussis toxin. Colonies were cultured on Matrigel without feeder cells, treated with pertussis toxin or cholera toxin, and imaged by time-lapse phase contrast microscopy. Within 12 h, pertussis toxin-treated colonies contracted inward, lost pseudopodia-like projections, and adopted a dense, aggregated morphology similar to that of mouse ES cell colonies (Figure 2, Supplemental Movie S1). Colonies treated with cholera toxin to constitutively activate G_s_ signaling, were not affected by treatment and, like control colonies, expanded over time.

### Pluripotent Colonies Require G_i_ Signaling to Heal a Scratch Wound Insult

**Figure 3:**
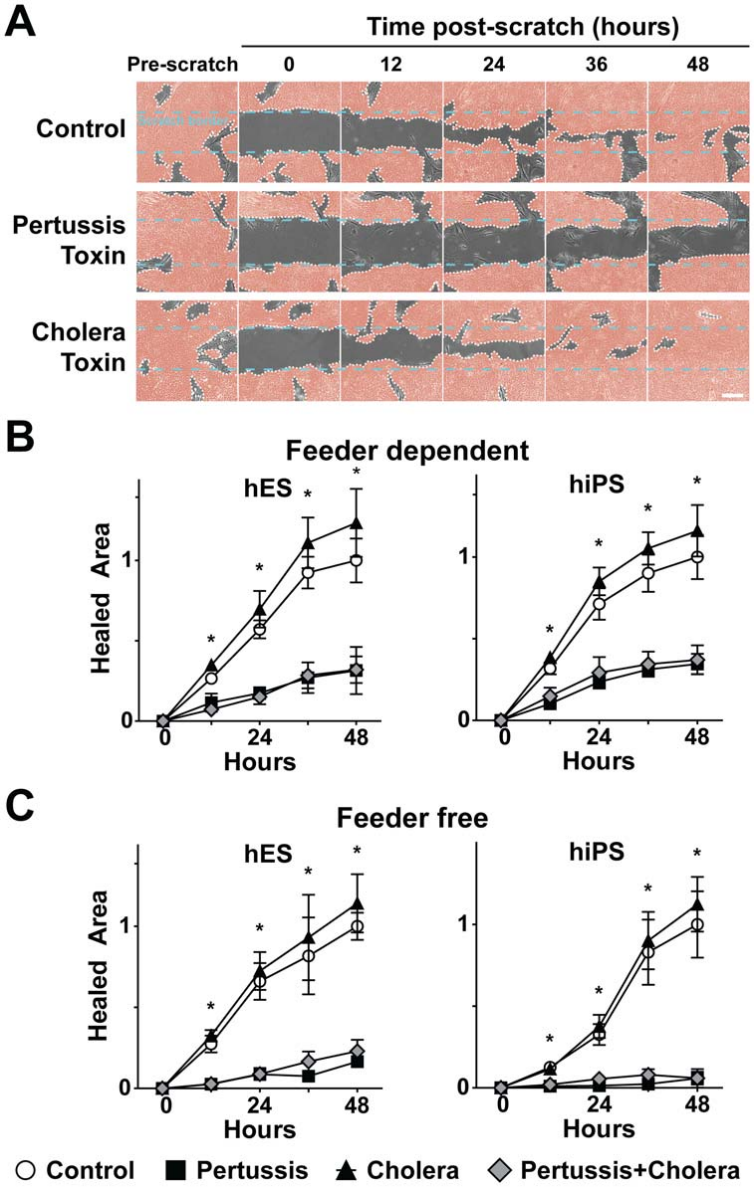
Pertussis toxin inhibits wound healing in human embryonic stem (hES) and human induced pluripotent stem (hiPS) colonies. **(A)** Time-lapse series shows that pertussis toxin, but not cholera toxin, attenuates pluripotent colony wound healing. Scale bar, 200 µm. **(B and C)** hES and hiPS cell colonies grown in the presence **(B)** or absence **(C)** MEF feeder cell lines exhibit significant reductions in colony wound healing after treatment with pertussis toxin, alone or together with cholera toxin, but not cholera toxin alone. Values are percent area healed relative to healing in control colonies. Error bars indicate the standard deviation. * *P < 0.05*.

To assess the functional role of GPCR signaling in pluripotent colony formation and, specifically, to determine if inhibition of G_i_ signaling disrupts endogenous organizing mechanisms, we used a scratch wound healing assay in which colony re-growth into a denuded area is monitored by time-lapse microscopy (Figure 3A). As we expected, hES cell or hiPS cell colonies treated with pertussis toxin were unable to reform after the scratch wound, whereas control colonies and colonies treated with cholera toxin healed completely within 48 h (Figure 3B). Wound healing was also reduced in pluripotent colonies cultured without mouse embryonic fibroblast (MEF) feeder cells (Figure 3C), suggesting that feeder cells are not required for the observed phenotype.

### Inhibition of G_i_ Signaling by Pertussis Toxin Disrupts Pluripotent Colony Organization Independently of Proliferation, Apoptosis and Pluripotency

**Figure 4:**
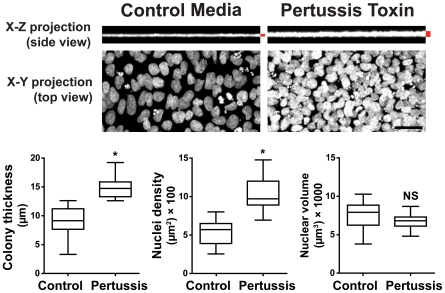
Disrupted pluripotent stem cell colony organization by pertussis toxin in human embryonic stem (hES) cells and human induced pluripotent stem (hiPS) cells. Three dimensional confocal projections of pluripotent colonies treated with pertussis toxin and stained with Hoechst show increased colony thickness and density, but no significant change in nuclear volume. Pluripotent stem cells treated with pertussis toxin aggregate and stack three dimensionally whereas control colonies maintain a monolayer conformation. Error bars indicate the minimum and maximum data points. Scale bar, 100 µm. * *P < 0.05*. NS = Not significant.

To investigate how pertussis toxin inhibited colony wound healing, we assessed the three-dimensional organization of pertussis toxin-treated colonies by analyzing Hoechst-stained nuclei by confocal microscopy. Colonies treated with pertussis toxin underwent re-organization and were significantly thicker and denser than control colonies (Figure 4), suggesting that inhibition of G_i_ signaling disrupts endogenous mechanisms that maintain human pluripotent stem cell colonies as characteristic flat monolayers. Nuclear volume did not differ significantly in treated and control colonies. Identical results were obtained in undifferentiated hES and hiPS cell colonies. Thus, inhibition of G_i_ signaling with pertussis toxin consistently changed both the morphology and organization of human pluripotent colonies resulting in thicker and denser colonies more closely resemble undifferentiated mouse ES cells.

**Figure 5:**
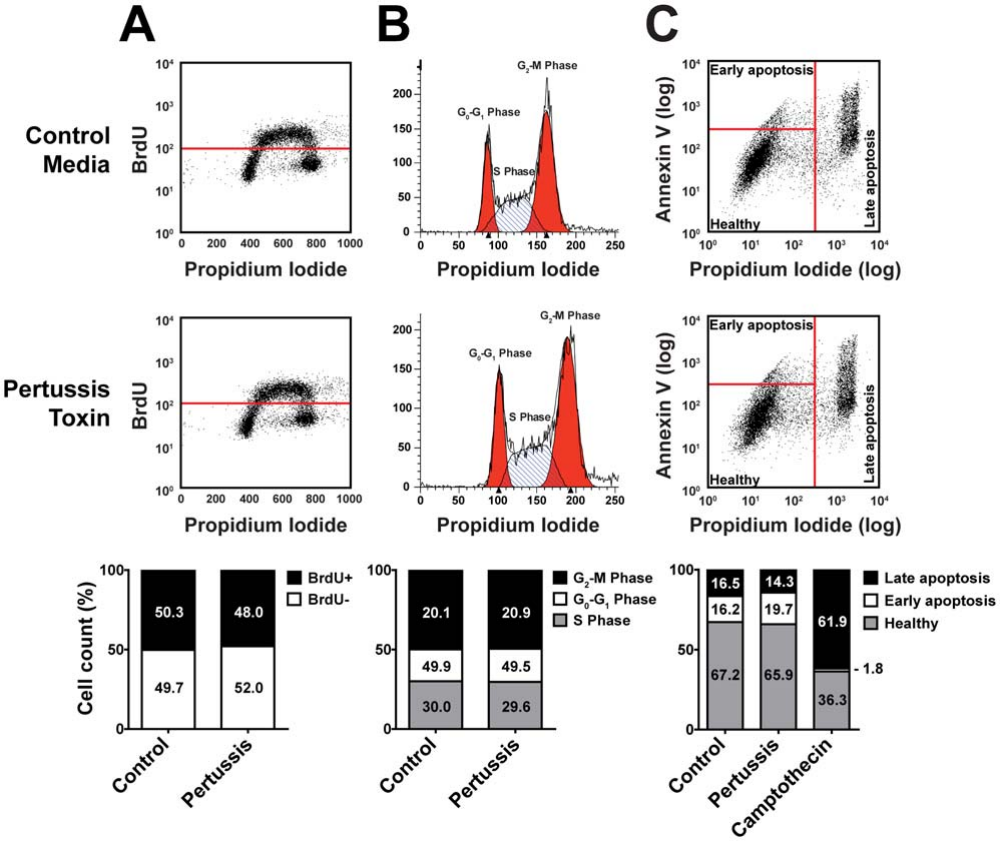
Proliferation and apoptosis are unchanged by pertussis toxin treatment in human embryonic stem (hES) cells and human induced pluripotent stem (hiPS) cells. **(A)** Pluripotent hES cell and hiPS cells colonies were treated with pertussis toxin or control medium and pulsed with BrdU for 2 h to detect proliferating cells. Representative flow cytometery data show no difference in BrdU incorporation between treatments: 48.0% of pertussis toxin-treated hES cells were BrdU positive vs. 50.3% of control cells. **(B)** Pluripotent hES cell and hiPS cells colonies were treated with pertussis toxin or control medium and stained with propidium iodide to model cell-cycle states. Representative flow cytometery histograms show no difference in number of cells in G_2_-M, G_0_-G_1_, or S phase between treatments. **(C)** To assess whether pertussis toxin affects apoptosis of pluripotent stem cells, colonies treated with pertussis toxin or control medium were stained with Annexin V, a marker of apoptosis, and analyzed by flow cytometry. Pluripotent hES cell and hiPS cells colonies were treated with either pertussis toxin, control or the pro-apoptotic agent camptothecin and stained with annexin V and propidium iodide. Representative flow cytometery data show no difference in BrdU incorporation between pertussis toxin and control treatments. Camptothecin induced apoptosis in 34.0% of pertussis toxin-treated cells and 32.7% of control cells.

**Figure 6:**
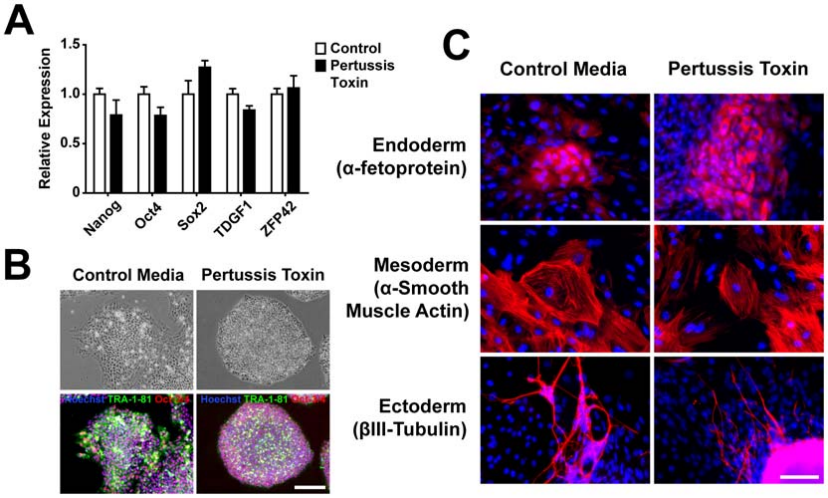
Pertussis toxin does not affect the maintenance of pluripotency pluripotent stem cell colonies or the ability to form all three embryonic germ layers. **(A)** Pertussis toxin does not affect the maintenance of pluripotency of hES or hiPS cells as assessed by the expression of the pluripotency markers Nanog, Oct4, Sox2, TDGF1, and ZFP42 by quantitative real-time PCR. **(B)** Immunocytochemistry of TRA-1-81 (green) and Oct-3/4 (red) also did not reveal differences between hES or hiPS cells treated with pertussis toxin and control treatments in pluripotent stem cell colonies. Nuclei were stained with Hoechst (blue). Scale bar, 200 µm. **(C)** hES or iPS cells treated with pertussis toxin formed embryoid bodies and differentiated into cell types of the all three embryonic germ layers as assessed by immunocytochemistry for α-fetoprotein (a marker of endoderm), α-smooth muscle actin (mesoderm) and βIII-tubulin (ectoderm). Scale bar, 100 µm.

Next, we determined whether pertussis toxin alters cell proliferation, apoptosis, or pluripotency, three variables that would confound interpretation of the morphological and organizational data. No significant differences in proliferation (Figure 5A, B) or apoptosis (Figure 5C) were observed in treated and control hES or hiPS cells, as assessed by 5-bromo-2′-deoxyuridine (BrdU) incorporation, cell-cycle modeling and annexin V staining. Pertussis toxin also did not affect the maintenance of pluripotency as assessed by the expression of pluripotency markers by quantitative real-time PCR (Figure 6A) or immunocytochemistry (Figure 6B). Nor did pertussis toxin affect the ability of colonies to differentiate into the three embryonic germ layers (Figure 6C) and develop mature phenotypes, such as rhythmically beating foci (Supplemental Movie S2). Thus, the effects of pertussis toxin cannot be explained by increased or decreased colony growth, viability or pluripotency, but rather represent a disruption in colony morphology and organization.

### hES cell and hiPS Cells Express GPCRs, Including Those of the G_i_ Signaling Family

**Figure 7:**
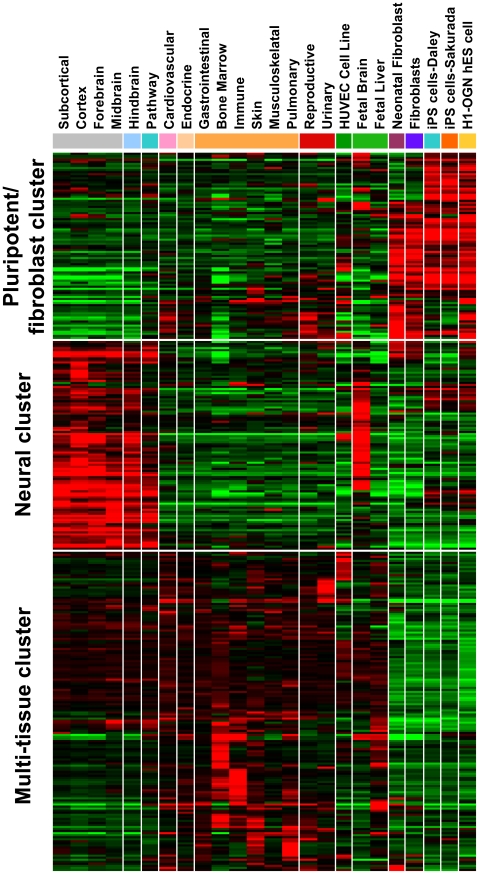
Gene expression clustering reveals enrichment of GPCRs in stem cells. A heat-map of microarray probesets aligning to predicted GPCRs across 100 somatic tissues and cell types, hES cells and hiPS cells. Relative expression differences were calculated relative to the mean expression of all tissues and cells for each probeset. The mean expression values of specific tissue groups (e.g., cardiovascular) were grouped to highlight the relative expression differences compared to hES cell and hiPS cells. Expression patterns reveal clustering of GPCRs according to pluripotent/fibroblast, neural or multi-tissue origin.

To place our findings in the context of endogenous GPCRs, we attempted to identify the GPCRs expressed in hES and hiPS cells. Identifying GPCR transcripts can be difficult because they are generally expressed at low levels, making DNA microarrays difficult to interpret. To identify G_i_-coupled GPCRs that might mediate G_i_ sensitivity in hES cells and hiPS cells, we comprehensively analyzed DNA microarray data for GPCR expression across many cell types and tissues. We constructed a meta-dataset of hES and hiPS cells, fibroblasts and 100 somatic normal tissues from publicly available data from the same DNA-microarray platform (U133 plus 2.0 array) [3], [29]. Comparison of GPCR expression across tissues yielded a large percentage of GPCRs expressed above background levels. Expression clustering of all GPCRs across tissues and cell lines revealed considerable enrichment in hES and hiPS cells with fibroblasts that were distinct from neural or other tissue origin (Figure 7).

Included in the list of GPCRs and seven-transmembrane receptors predicted to be expressed or enriched were those implicated in embryonic stem cell function, such as CXCR4 [30], CNR1 [31],VIPR2, FZD5, FZD7, and FZD10 [32]. Filtering GPCRs by evidence of adenylate cyclase inhibition identified 25 unique GPCRs potentially expressed in pluripotent cells (Table 1). Although this global analysis only infers expression and does not provide definitive proof, it suggests that many GPCRs are expressed in human pluripotent stem cells and provides support of our initial hypothesis and directions for future study, including genome-wide sequencing of cellular mRNAs (RNA-seq) and pharmacological testing of each candidate receptor.

**Table 1:** 25 G_i_-coupled GPCRs are potentially expressed in pluripotent cells.

Symbol	Description	iPS/ES p-value	iPS/ES fold difference	GPCR-coupling
ADORA3	adenosine A3 receptor	8.40E-06	−1.84	Gi
ADRA2A	adrenergic, alpha-2A-, receptor	5.37E-05	2.82	Gi
ADRA2C	adrenergic, alpha-2C-, receptor	2.18E-06	1.37	Gi
CNR1	cannabinoid receptor 1 (brain)	9.54E-05	2.71	Gi
CNR2	cannabinoid receptor 2 (macrophage)	1.20E-02	−1.20	Gi
CXCR4	chemokine (C-X-C motif) receptor 4	8.05E-03	1.72	Gi
DRD3	dopamine receptor D3	1.32E-06	1.20	Gi
DRD4	dopamine receptor D4	1.27E-06	2.08	Gi
EDG2	endothelial differentiation, lysophosphatidic acid G-protein-coupled receptor, 2	4.72E-03	2.05	Go,Gq,G13,G12,Gi
EDG4	endothelial differentiation, lysophosphatidic acid G-protein-coupled receptor, 4	1.96E-08	2.28	Go,Gq,G13,G12,Gi
EDG7	endothelial differentiation, lysophosphatidic acid G-protein-coupled receptor, 7	9.33E-11	2.45	Go,Gq,Gi
EDG8	endothelial differentiation, sphingolipid G-protein-coupled receptor, 8	3.70E-08	1.86	G12,Gi
FFAR2	free fatty acid receptor 2	1.56E-03	1.25	Gq,Gi
FFAR3	free fatty acid receptor 3	6.40E-05	1.37	Gq,Gi
GABBR1	gamma-aminobutyric acid (GABA) B receptor, 1	5.96E-05	1.74	Gq,Gi
GABBR2	gamma-aminobutyric acid (GABA) B receptor, 2	9.77E-06	4.58	Gq,Gi
GALR2	galanin receptor 2	4.71E-07	1.72	Gq,Gi
GRM2	glutamate receptor, metabotropic 2	7.85E-08	1.73	Gi
GRM4	glutamate receptor, metabotropic 4	1.13E-02	1.21	Gi
HTR1B	5-hydroxytryptamine (serotonin) receptor 1B	1.78E-03	1.22	Gi
HTR1D	5-hydroxytryptamine (serotonin) receptor 1D	1.07E-03	1.83	Gi
MCHR1	melanin-concentrating hormone receptor 1	1.36E-04	1.49	Gq,Gi
NPY5R	neuropeptide Y receptor Y5	1.47E-04	1.41	Gi
OXER1	oxoeicosanoid (OXE) receptor 1	1.98E-04	−1.23	Go,Gi
P2RY13	purinergic receptor P2Y, G-protein coupled, 13	2.06E-04	−1.36	Gi

The GPCRs were identified by filtering the microarray dataset (Supplemental Dataset S1) for G_i_-coupled GPCRs likely expressed in hES or hiPS cells (p-value < 0.05 and mean fold difference ≥2).

## Discussion

**Figure 8:**
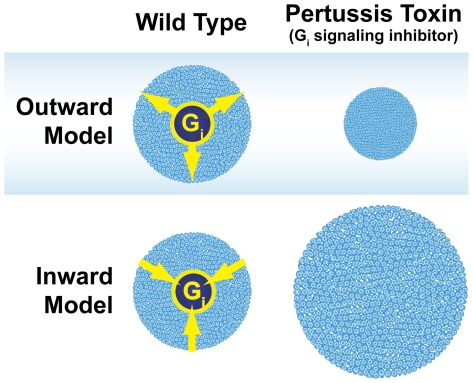
Model of organization of pluripotent stem cell colonies. G_i_ signaling may control pluripotent stem cell morphology and organization through density-sensing that outwardly organizes pluripotent stem cell colonies in a homogenous monolayer. Disruption of G_i_ signaling with pertussis toxin results in colonies that are contracted inward, densely aggregated and stacked. An alternative model in which G_i_ signaling mediates inward organization of pluripotent stem cells was not supported by the data.

This study shows that the colony morphology characteristic of pluripotent hES and hiPS cells is maintained through a pertussis toxin-sensitive mechanism downstream of G_i_-coupled GPCRs. Wild-type colonies were discrete and radial with flat, monolayer organization. Absence of this characteristic morphology and organization usually suggests a lack or loss of pluripotency in human pluripotent cell cultures [2], [7]. G_i_ signaling may specifically mediate the morphology and organization of pluripotent stem cell colonies by outwardly regulating the density of cells within the colony. We refer to this as the “outward model” of pluripotent stem cell organization (Figure 8).

Human pluripotent stem cells organize as a flat monolayer of cells that tightly adhere to one another. The Rho-Rock-myosin signaling axis is required to maintain this strong cell-cell adhesion [33]. Inhibition of Rock renders hES cell colonies more resistant to enzymatic disassociation [34] and survival as a single-cell suspension [35]. Although the Rho-Rock-myosin pathway is known to induce changes in hES cell colony morphology [33], activation of this pathway is known to be independent of G_i_ signaling and is insensitive to pertussis toxin in somatic cells [36], [37]. GPCRs coupled to the G proteins G_q_, G_11_, G_12_, and G_13_ activate Rho in a pertussis toxin-independent manner [38]–[40], so the Rho-Rock-myosin pathway will need to be closely examined as we extend our studies to these G protein pathways. The exact molecular mechanism for G_i_-induced morphology change is unknown and there are likely other ways that the GPCR superfamily could activate changes to cellular morphology. We anticipate that there will be multiple GPCR pathways that induce morphology change that work in parallel with the G_i_ signaling pathway. The morphology change that we observe with pertussis toxin is also likely to alter the expression and function of adhesion molecules such as E-cadherin, a key regulator of ES cell adhesion [41]. Unfortunately, we do not at this time have a complete annotation of all cell surface adhesion molecules involved in this process. These genes are often expressed at low levels where DNA microarray data is not accurate. We are currently embarking on deep sequencing of hES and hiPS cells to gain a more robust list of the cell surface molecules that can be probed for involvement in human pluripotent cell morphology.

Our findings indicate that G_i_ signaling orchestrates the organization of human pluripotent stem cell colonies as an interconnected monolayer of cells. G_i_ signaling may maintain pluripotent stem cell colony morphology by outwardly organizing cells much like the springs anchoring the stretched fabric of a trampoline (Figure 8). The remarkable wound healing exhibited by wild-type pluripotent colonies suggests that colonies can detect the wounded area and actively reform to maintain colony integrity. Disrupting G_i_ signaling with pertussis toxin blocks the outward forces so that the colonies cannot re-enter the denuded area of the scratch assay, thereby inducing a morphological and organizational change.

Inhibition of G_i_ signaling with pertussis toxin converted the monolayer colony characteristic of wild-type pluripotent cells into a multilayered colony. The monolayer colony is a hallmark of human pluripotent cells, first described for hES cells [5] and later for hiPS cells [2]. These results suggest that a G_i_-coupled receptor actively participates in maintaining the morphology of human pluripotent colonies. Characteristic colony morphology serves as an important initial screen during the production of hiPS cells [2], [7]. Insights into how the morphology and organization of pluripotent colonies are regulated will thus be important to understanding the molecular mechanisms of somatic cell reprogramming. We hypothesize that similar G_i_-coupled GPCRs are involved in pluripotent colony formation and maintenance during iPS cell induction. These mediators of iPS cells formation may provide clues to identify factors important for the production of hiPS cells free of genetic modification.

G_i_ signaling may also play a role in the long observed difference between mouse and human pluripotent colonies. In contrast to monolayer human ES colonies, mouse ES cells form multilayered colonies that are clearly pluripotent as they satisfy the most stringent criteria for pluripotency including tetraploid complementation [42]. In view of the significant differences in colony morphology, some investigators have wondered whether human ES cells are truly as pluripotent as mouse ES cells. Interestingly, mouse ES cells treated with pertussus toxin exhibit a phenotype that is similar to that in hES and hiPS cells but attenuated (data not shown). The mouse cell phenotype may be harder to elicit because they are already retracted in their basal state. This suggests that mouse and human pluripotent stem cell colonies share a common G_i_-mediated mechanism for colony organization. The difference in wild-type colony morphology between mouse and human pluripotent cells may reflect differential amounts of G_i_-coupled GPCR signaling in pluripotent colonies. This hypothesis can be tested in future studies by directly stimulating G_i_-coupled GPCRs in mouse and human ES cells using engineered receptors such as RASSLs (receptor activated solely by a synthetic ligand) [43].

By analyzing a large tissue and cell line meta-dataset, we identified GPCRs, including G_i_-coupled receptors, with evidence of expression in human pluripotent stem cells. Such an analysis has several drawbacks over more quantitative measures (e.g., transcript sequencing, protein detection), but nevertheless provides a preliminary candidate list for identifying mediators of the G_i_ sensitivity of hES and hiPS cells. Manipulating the processes by which pluripotent colonies form and organize may also be critical for therapeutic applications. As hiPS cell technology matures as a viable source for cell-based therapeutics and regenerative medicine, controlling the multi-cellular morphological and organizational characteristics of pluripotent stem cells *ex vivo* will become increasingly important. GPCRs and their downstream signaling pathways are attractive targets for study in human pluripotent stem cells.

## Materials and Methods

### Cell Culture

Pluripotent hES cells (H9, WiCell) with fewer than 50 passages and hiPS cells derived from adult human dermal fibroblasts (Cell Applications) were generated as described [2]. Undifferentiated hES cells were cultured as described [2], [44] with minor modifications. Briefly, cells were cultured in knockout Dulbecco's modified Eagle's medium/F-12 (Invitrogen, Carlsbad, CA) supplemented with 1 mM L-glutamine, 20% knockout serum replacement medium (Invitrogen), 1 mM sodium pyruvate (Invitorgen), 0.1 mM nonessential amino acids (NEAA, Invitrogen), 0.5% penicillin and streptomycin (UCSF Cell Culture Facility), 0.1 mM beta-mercaptoethanol (Sigma), and 10 ng/ml full-length basic fibroblast growth factor (Invitorgen). Cells were grown in MEF-conditioned medium in six-well plates (Corning, catalog no. 3506) coated with growth factor-reduced Matrigel (BD Bioscience) with MEF-conditioned medium or on a feeder layer of irradiated SNL MEFs [45]. Matrigel was prepared in cold Knockout DMEM at 1∶100 dilution and allowed to coat the culture plates for at least 45 min at room temperature. Excess Matrigel was removed, and the plates were washed once with Dulbecco's phosphate-buffered saline (DPBS) immediately before plating of cells. SNL MEFs were gamma-irradiated with 3000 rads (30 Grays) and plated at 10^4^ cells per cm^2^. All cells were passaged after enzymatic digestion with Accutase (Millipore) approximately every 7 days as described [46]. For passage, cells were washed twice with DPBS and treated with a minimal amount of Accutase for 1 min at room temperature. Accutase was removed, and the culture was gently washed twice with DPBS (Ca^2+^- and Mg^2+^-free). Cells were harvested by gentle cell scraping and split at a ratio of 1∶4 to 1∶6 and cultured for 24 h with 10 µM ROCK inhibitor (Y-27632, Calbiochem) as described [34]. Cells were routinely tested for mycoplasma (Mycoplasma Plus PCR Primer Set, Stratagene).

### Toxin Treatment

Pertussis toxin from *Bordetella pertussis* (Sigma) was prepared in water with 2.5 mg/ml bovine serum albumin (BSA) (Sigma) to a stock concentration of 50 µg/ml. Cholera toxin from *Vibrio cholerae* (Sigma) was prepared in water to a stock concentration of 10 mg/ml. Pluripotent stem cells were treated with pertussis toxin (final concentration, 200 ng/ml) or cholera toxin at the final concentration of 10 µg/ml for 2 h in Knockout DMEM/F-12 medium supplemented with 0.25% BSA, 1 mM L-glutamine, 1 mM sodium pyruvate, 0.1 mM NEAA, 0.5% penicillin and streptomycin, and 0.1 mM beta-mercaptoethanol, followed by 1∶1 addition of pluripotent stem cell culture medium containing 20% Knockout Serum Replacement medium for 12 h. Controls were treated in the same fashion except that water containing 2.5 mg/ml BSA was used instead of toxin. The medium was changed daily with human pluripotent medium described previously and maintained under standard pluripotent conditions throughout the assay.

### Time-Lapse Microscopy

Control and pertussis toxin-treated colonies were imaged with a Carl Zeiss Axio Observer Z1 and AxioVision version 4.7 software (Carl Zeiss) and configured with a linearly scaled stage and an XL S incubation unit (Carl Zeiss) to maintain physiological atmospheric conditions at 37°C and 5% CO_2_ with humidification. Time-lapse images were captured with an EC Plan-Neofluar 5×/0.16 M27 objective with 1.6 Optivar (Carl Zeiss) every 5 or 10 min and exported to Adobe Premiere CS3 software for compilation, analysis and video output.

### Scratch Wound Healing Assay

Pluripotent hES and hiPS cells were cultured as discrete colonies to 50–75% confluency and treated with control medium, pertussis toxin, or cholera toxin as described above. A linear scratch wound was placed across the culture with a 200 µl LTS pipette tip (Rainin) and washed once with Knockout DMEM/F12 to remove debris. The scratched cultures were serially imaged by phase-contrast microscopy or prepared for time-lapse imaging and cultured in pluripotent stem cell culture medium for the duration of the assay. Analysis was performed with Adobe Photoshop CS3 by manually masking the boundaries of the scratched area and quantifying the area of colony healing over time using the Photoshop Extended Measurement feature. Data are reported as the percentage of healed area relative to that in control colonies. This assay assumes that proliferation and apoptosis are unchanged by the treatment parameters. Thus, controlling for these potential confounders is important to validate any results.

### Confocal Microscopy

Control and pertussis toxin-treated colonies were fixed with 4% paraformaldehyde for 15 min, stained with Hoechst stain (Invitrogen; 1∶2000) for 10 min, washed twice with DPBS, and immediately imaged on a Carl Zeiss LSM510 Meta Confocal Microscope with a Plan-Neofluar 25×/0.8 multi-immersion objective (Carl Zeiss). Confocal projections were compiled with MetaMorph version 5 (Molecular Devices) and Zen LE (Carl Zeiss) software. Colony thickness was measured on confocal projections with MetaMorph. Automated nuclei counting of confocal images was performed with ImageJ (http://rsbweb.nih.gov/ij) using a modification of the 3D Object Counter plugin (http://rsbweb.nih.gov/ij/plugins/track/objects.html; script available upon request). Nucleic volume was calculated with ImageJ and a modification of Voxel Counter plugin (http://rsbweb.nih.gov/ij/plugins/voxel-counter.html; script available upon request).

### Proliferation Assay

Colonies maintained on Matrigel and MEF-conditioned medium were treated with control medium or pertussis toxin, cultured in pluripotent conditions for 24 h after treatment and pulsed with 100 µM BrdU for 2 h. Cells were cultured for an additional 12 h in pluripotent conditions and harvested with Accutase as described above, mechanically dispersed to a single-cell suspension, and washed by repeated centrifugation. Cells were fixed with 70% ethanol and stored at −20°C until further processing. Cells were pelleted and washed twice with wash buffer (10 mM HEPES, pH 7.4, 150 mM NaCl, 4% FBS) and incubated in 0.5 ml of 2 M HCl +0.5% FBS for 20 min at room temperature. Cells were washed again in wash buffer, pelleted, and resuspended in 100 mM sodium tetraborate (Sigma) for 2 min at room temperature. Cells were again washed with wash buffer and stained with anti-BrdU-Alexa 488 flourphore prepared 1∶1000 in DPBS and incubated for 30 min at room temperature. Stained cells were washed twice with wash buffer, pelleted, and co-stained with 10 µg/ml propidium iodide with 1 mg/ml RNase A in DPBS for 30 min at room temperature. Stained cells were filtered and counted on a FACSCalibur flow cytometer (BD Biosciences) and analyzed by FlowJo software (Tree Star). DNA distributions were analyzed with ModFit (Verity Software House) to determine the proportions of pluripotent stem cells in G_0_-G_1_, S, and G_2_-M phases of the cell cycle.

### Apoptosis Assay

Colonies maintained on Matrigel and MEF-conditioned medium were treated with control medium or pertussis toxin as described above and cultured in pluripotent conditions for 24 h. Camptothecin (Sigma), a pro-apoptotic agent, was used as a positive control at 5 µM in DMSO and incubated for 5 h. Cells (5×10^5^) were harvested as described above and mechanically dispersed to a single-cell suspension by trituration. Cells were stained with the Annexin V-FITC apoptosis detection kit (BD Pharmingen) according to the manufacturer's protocol with minor modifications. Briefly, cells were resuspended in binding buffer at 1×10^6^ cells/ml and stained with Annexin V and propridium iodide for 15 min, diluted 1∶1 with binding buffer, filtered, and counted immediately on a FACScalibur flow cytometer. The data were analyzed with FlowJo software version 8.8.6.

### Targeted Gene Expression Assay

ES cells or EBs were harvested in Trizol (Invitrogen) for total RNA isolation. For mRNA qRT-PCR, 2 µg of total RNA from each sample was reversed transcribed with Superscript III (Invitrogen). cDNA (10 ng) was used for subsequent PCRs, performed in triplicate on an ABI 7900HT instrument (Applied Biosystems) using Taqman primer probe sets (Applied Biosystems) for each gene of interest and a GAPDH endogenous control primer probe set for normalization. Each qRT-PCR was performed on three different experimental samples; representative results are shown as fold expression relative to undifferentiated and control hES cells. Error bars reflect one standard deviation from the mean of technical replicates.

### hES Cell and hiPS Cell Differentiation

hES cell and hiPS cells were grown to near confluency. Differentiation was performed as described [44]. Briefly, differentiation was initiated by embryoid body (EB) formation on day 0 by treatment with 1 mg/ml collagenase IV followed by two rinses with DPBS to remove any residual MEFs. The collagenase IV-treated colonies were dispersed by gentle mechanical pipette trituration into cell aggregates of 500–800 cells in differentiation medium (Knockout DMEM/F-12 supplemented with 20% fetal bovine serum (FBS; Hyclone), 1 mM L-glutamine, 1 mM sodium pyruvate, 0.1 mM NEAA, 50 U/ml penicillin, 50 mg/ml streptomycin, and 0.1 mM beta-mercaptoethanol). Aggregates were immediately re-plated on low attachment plates (Corning). The medium was replaced with fresh medium once on day 4, and the EBs were transferred onto 0.1% gelatin-coated 10 cm^2^ culture dishes and followed for up to 24 additional days; half the medium was replaced with fresh medium every other day.

### Immunocytochemistry

Cells were fixed with 4% paraformaldehyde in PBS for 10 min at room temperature, washed with PBS, and washed twice in 0.1% Triton X-100 (Sigma) in PBS. Cells were blocked with 5% normal goat serum (Sigma) in PBS containing 0.1% Triton X-100 for 1 h at room temperature. The primary antibodies were Oct-3/4 (1∶1000, MAB1759, R&D Systems), TRA-1-81 (1∶50, MAB4381, Chemicon), α-fetoprotein (1∶100, MAB1368, R&D Systems), α-smooth muscle actin (prediluted, DAKO), and βIII-tubulin (1∶100, CB412, Chemicon). The secondary antibodies were cyanine 3 (Cy3) -conjugated goat anti-rat IgM (1∶500, Jackson Immunoresearch), Alexa555-conjugated goat anti-mouse IgG (1∶500, Invitrogen), Alexa555-conjugated goat anti-mouse IgM (1∶500, Invitrogen), and Alexa488-conjugated goat anti-mouse IgM (1∶500, Invitrogen). Nuclei were stained with 1 mg/ml Hoechst 33342 (Invitrogen). Stained cells were imaged by epiflourescence on a Zeiss AxioObserver with LD Plan-Neofluar 20×/0.4 Korr objective and AxioVision software. Images were exported to Adobe Photoshop CS3 for compilation.

### Statistical Analysis

All experiments were performed a minimum of three times. Two-sample *t* tests (equal variance) were used to analyze the significance of difference for apoptosis assays. *P* < 0.05 was considered statistically significant. Standard deviations were calculated for the scratch wound healing, Annexin V, and BrdU incorporation assays.

### Gene Expression Microarray Analysis

For the microarray meta-dataset analysis, all Affymetrix CEL files were downloaded from NCBI Gene Expression Omnibus (http://www.ncbi.nlm.nih.gov/geo) for the U133 plus 2.0 array platform from published datasets. This dataset consisted of normal adult human tissue samples from the human body index compendium study (GSE7307), hES cells, hiPS cells, and fibroblast lines (GSE9709 and GSE9832) [3], [29]. For all datasets, one to three arbitrary biological replicates per tissue were used (based on availability). All data was co-normalized in the program AltAnalyze [47] with the RMA algorithm, producing mean expression values for each group of biological replicates.

### GPCR Annotations

Probesets from this dataset were linked to GPCR annotations and phenotypes from UniProt and the Mouse Genome Informatics databases, respectively, using a custom Python script. To produce GPCR coupling annotations, this script uses a custom heuristic to identify the G-protein alpha protein sub-type (G_αi_, G_αq_, G_αs_, G_α11_, G_α12_, G_α13_, G_α olf_, G_α taste_), based on the UniProt curated description field (script available upon request).

### Determining Expression of GPCRs

To predict whether GPCRs in hES or hiPS cells are expressed, sufficiently above background we compared expression in these cells to other tissues. Typically Affymetrix Absent-Present (AP) calls are sufficient to identify GPCRs that are predicted to be expressed. However, since AP calls varied considerable variability between replicates (data not shown), we used an alternative method. Each hiPS cell and hES cell line was compared to the 10 lowest expressing tissues/cell lines (excluding hiPS cell and hES cell). All fibroblast cell lines were combined into a single group. A *t* test was performed on the 10 lowest expressing tissues for each GPCR relative to the combined hiPS cell and hES cell lines. GPCRs with a p-value < 0.05 and a mean fold change ≥1.5 were considered to be expressed. GPCR expression clustering was performed for all tissues and cell lines using the program HOPACH (hierarchical ordered partitioning and collapsing hybrid), with uncentered correlation distance, relative to the mean expression of all tissues and cells [48], [49]. All data from this analysis are provided as supplemental data (Supplemental Dataset S1 and Supplemental Dataset S2).

## Supporting Information

Movie S1 Time-lapse movie of colony morphology rearrangement by pertussis toxin in human embryonic stem (hES) cells. A hES cell colony cultured on Matrigel under feeder-free conditions contracts inward, loses pseudopodia-like projections and adopts a dense, aggregated morphology within 12 h after pertussis toxin treatment. Colonies were treated with control medium or cholera toxin formed monolayers. Identical observations were made in human induced pluripotent stem (hiPS) cells. Movie frames were captured at 5 min intervals. Scale bar, 200 µm.
(0.90 MB MOV)

Movie S2 Time-lapse movie of beating colonies formed by both control and pertussis toxin-treated human induced pluripotent stem (hiPS) cell colonies. Treatment with pertussis toxin did not affect differentiation of hiPS cells into embryoid bodies to yield beating foci. Efficiencies of deriving beating foci were similar between treatments. Identical observations were made in human embryonic stem (hES) cells. Video was captured in real-time. Scale bar, 200 µm.
(0.72 MB MOV)

Dataset S1 Excel worksheet of microarray analyses.
(0.17 MB XLS)

Dataset S2 Raw dataset used to perform microarray analyses.
(1.56 MB XLS)
